# Behaviour of aerosols and their role in the transmission of SARS‐CoV‐2; a scoping review

**DOI:** 10.1002/rmv.2297

**Published:** 2021-10-01

**Authors:** José Miguel Robles‐Romero, Gloria Conde‐Guillén, Juan Carlos Safont‐Montes, Francisca María García‐Padilla, Macarena Romero‐Martín

**Affiliations:** ^1^ Department of Nursing University of Huelva Huelva Spain; ^2^ Ministry of Education, Government of Spain, Delegation of Huelva Huelva Spain; ^3^ Hospital Universitario Virgen Macarena Sevilla Spain

**Keywords:** aerosol, airborne transmission, bioaerosol, coronavirus, Covid‐19, droplet, SARS‐CoV‐2, social distance, ventilation

## Abstract

Covid‐19 has triggered an unprecedented global health crisis. The highly contagious nature and airborne transmission route of SARS‐CoV‐2 virus requires extraordinary measures for its containment. It is necessary to know the behaviour of aerosols carrying the virus to avoid this contagion. This paper describes the behaviour of aerosols and their role in the transmission of SARS‐CoV‐2 according to published models using a scoping review based on the PubMed, Scopus, and WOS databases. From an initial 530 references, 9 papers were selected after applying defined inclusion criteria. The results reinforce the airborne transmission route as a means of contagion of the virus and recommend the use of face masks, extending social distance to more than 2 metres, and natural ventilation of enclosed spaces as preventive measures. These results contribute to a better understanding of SARS‐CoV‐2 and help design effective strategies to prevent its spread.

**Abbreviations**
Covid‐19: Coronavirus disease 19
FFP: Filtering Face Piece
HVAC: Heating Ventilation Air Conditioning
SARS‐CoV‐2: Severe Acute Respiratory Syndrome Coronavirus 2
WHO: World Health Organisation
WOS: Web of Science

## 1 INTRODUCTION

Coronavirus disease 2019 (Covid‐19) has triggered an unprecedented global health crisis. At the end of June 2021, the total number of cases worldwide rose to 184,572,371, of which 3,997,640 died.^1^ Although the behaviour and development of the disease varies, the Centre for Disease Control and Prevention estimates a mortality rate between 0.7% and 5.7%.^2^ The unknown and highly contagious nature of the disease requires extraordinary measures for its treatment and containment.

The disease is caused by a virus, Severe Acute Respiratory Syndrome Coronavirus 2 (SARS‐CoV‐2), which is considered one of the most contagious, as it is rapidly transmitted from person to person by close contact.^3^ In general, respiratory viruses are transmitted through three ways: contact transmission, when someone comes into direct contact with an infected person or touches a surface that has been contaminated, and then touches the eyes, nose, or mouth. Secondly, through droplet transmission, by means of inhalation or direct inoculation through the eyes, nose, or mouth of respiratory droplets, both large and small, containing the virus, which would occur when near an infected person. Thirdly, through airborne transmission, by inhalation through the upper or lower airways of droplets and smaller particles that are suspended in the air for longer distances and times than droplet transmission.^4^

SARS‐CoV‐2 spreads primarily through the respiratory particles that infected people emit when talking, coughing, breathing, or sneezing, wrapped in mucus or saliva forming droplets. The behaviour of these droplets in the air depends on their size; larger droplets precipitate before evaporating, splashing their nearby environment.^5^ When these drops are larger than 5–10 μm in diameter, their emission has a range of 1 metre, so an infected person could infect another healthy person with whom they have close contact.^6^ In addition, it can also be spread by coming in contact with the contaminated belongings and environment of an infected person, as the virus can persist on inanimate surfaces for days.^5^ At the beginning of the pandemic, this pathway, surface transmission, was considered the main route of spreading of the virus, and airborne transmission was thought unlikely.^4^ Consequently, and in the absence of a vaccine or specific treatment for Covid‐19, prevention measures focused on mask use, social distancing and confinement, hand hygiene, and disinfection of surfaces.^7^

However, the role of aerosols in the transmission of SARS‐CoV‐2 is increasingly relevant. Aerosols are dispersed liquid or solid particles suspended in the air, which may contain microorganisms, in which case they are called bioaerosols.^5^ Aerosols have a size <5 μm and, being smaller and lighter than droplets, do not precipitate in the proximity of the infected person, but can remain in the air for an extended period of time.^8^ Respiratory droplets are mainly composed of water with a solid particle inside such as microorganisms. The effect of evaporation, conditioned by relative humidity, temperature, and air speed, causes the droplets to quickly become aerosols up to a certain size, carrying a considerable effective viral load.^9^ Aerosols with viral load can directly enter the host's airways by inhalation, thus spreading the disease.^10^

**FIGURE 1:**
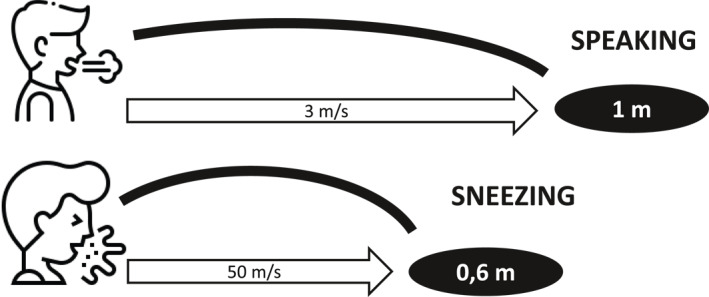
The emission of particles through the mouth or nose

Breathing, talking, coughing, and sneezing generate drops and sprays sliding from one individual to another. These actions mainly generate microparticles, except for sneezing, which provokes the emission of large drops with central tendency.^11^ The least particle‐producing actions are breathing, followed by talking and then coughing.^12^ Singing increases emission rates and has been associated with cases of SARS‐CoV‐2 transmission in enclosed spaces.^13^ The emission of particles through the mouth or nose depends on the initial speed with which they are dispatched. A sneeze can expel particles at high speed (about 50 m/s), but these dissipate quickly at a short distance (5 m/s at more than 0.6 m). When speaking, particles are emitted with a lower initial speed (3 m/s), though the airflow field has a range of 1 m (Figure 1).^12^

The transmission mode affects whether the infection will be beginning in the upper or lower respiratory tract and, consequently, the severity and evolution of the disease.^10^ Small aerosols are more susceptible to being inhaled deep into the lung, which would cause an infection in the alveolar tissues of the lower respiratory tract, while droplets become trapped in the upper respiratory tract.^14^ The port of entry of SARS‐Cov‐2 in humans is the angiotensin‐2 converting enzyme, that is present in type II pneumocytes of the lung and hair cells of the nasal membrane. From the biological point of view, airborne transmission of the virus is quite plausible.^12^

Between 80% and 90% of the particles emitted in respiratory activities by an infected person are aerosols, with a size less than 1 μm in diameter.^8^ In addition, the particles emitted by the cough of an infected person rapidly decrease in diameter, to half of their initial volume due to the loss of water by evaporation.^8^ That is, an infected person is surrounded by a nebula of aerosols generated by their own breathing. Studies have described how pathogens are most often found in small particle aerosols (<5 μm in diameter) that are airborne and breathable.^4^ Therefore, a large number of infectious particles with viral load circulating the air could be deposited in the nasal airways, and even host exposure can occur hours later and in the absence of the infected person.^8^

An aerosol is a particle that is subject to the aerodynamics of the gas in which it is found, so environmental conditions such as humidity, temperature, and ventilation can accelerate the transmission of aerosols, thus contributing to the spread of the disease.^15^ Aerosols with a small concentration of the virus, in poorly ventilated spaces, combined with low humidity and high temperature can be highly contagious.^16^ There have been cases of transmission between people more than 2 m away in enclosed spaces with little ventilation and after exposure to an infected person for more than 30 min^4^ Air conditioning and ventilation mechanisms increase the propagation distance of exhaled particles. Heating Ventilation Air Conditioning (HVAC) systems establish recirculation and ventilation circuits that can transport the aerosol from their point of origin to all HVAC areas. Thus, although the particles may reach further, the concentration will be lower due to dilution and filtration.^12^

On the other hand, although the main source of aerosols and droplets is the emission by patients themselves, there are other scenarios that can generate infectious particles, such as medical procedures, surgeries, tap water, and toilet flushes.^17^ According to the WHO, there are hospital procedures that could generate aerosols and therefore be a plausible route of transmission of the virus, such as endotracheal intubation, bronchoscopy, open aspiration, administration of nebulised treatment, manual ventilation prior intubation, turning the patient to prone position, disconnection of the patient from the ventilator, ventilation with non‐invasive positive pressure, tracheostomy, and cardiopulmonary resuscitation.^18^ This route of transmission poses an added risk to healthcare staff, who already suffered from close contact exposure associated with the care of infected patients.

For all these reasons, preventive measures against the coronavirus disease should include containment by airborne transmission. It is necessary to know the role of aerosols in spreading the disease in order to design effective preventive measures to protect the population.

The objective of this paper is to describe the behaviour of aerosols and their role in the transmission of SARS‐CoV‐2 according to published models.

## 2 MATERIALS AND METHODS

A scoping review was conducted following the methodology proposed by Arksey & O'Malley.^19^

### 2.1 Identifying the research question

The research question this review intended to answer was: How do aerosols emitted by respiratory activity behave in ambient air? How might this behaviour affect the transmission of SARS‐CoV‐2?

### 2.2 Identifying relevant studies

The PubMed, Scopus, and WOS databases were consulted. In addition, the reference lists of the most relevant articles were consulted. Search terms related to virus and disease, contaminating particles and route of transmission were selected. The search strategy used was (“Covid‐19” OR “SARS‐COV‐2” OR coronavirus) AND (aerosol OR droplet) AND (ventilation OR “airborne transmission”). The search was conducted between the months of January and February, 2021.

### 2.3 Study selection

Articles from experimental models describing the behaviour of aerosols in ambient air published between 2020 and 2021, in English or Spanish, were included in the present review. Two reviewers independently checked the references initially obtained in the search. After eliminating duplicates, the reviewers analysed the title and abstract to select papers according to the inclusion criteria. In case of doubt or disagreement, an attempt was made to make a decision by consensus, with a third researcher intervening when necessary. The shortlisted papers were analysed in full text to identify those that provided relevant information to the research question.

### 2.4 Charting the data

For data extraction, an analytical‐narrative method was followed to synthesise and interpret the information, grouping and reorganising the data by following the key points and main subjects (Arksey & O'Malley, 2005). The data were classified in tables specifically designed for this purpose, in the following sections: authorship, year of publication, model used in the study, measured variables, results, and recommendations. The whole process was carried out independently by two researchers who agreed on the extracted data. The intervention of a third researcher was not necessary to resolve discrepancies.

### 2.5 Collating, summarising, and reporting the results

The main findings and recommendations were summarised following an analytical‐descriptive procedure. The content analysis led to three main subjects: particle behaviour, containment measures, and ventilation.

## 3 RESULTS

**FIGURE 2:**
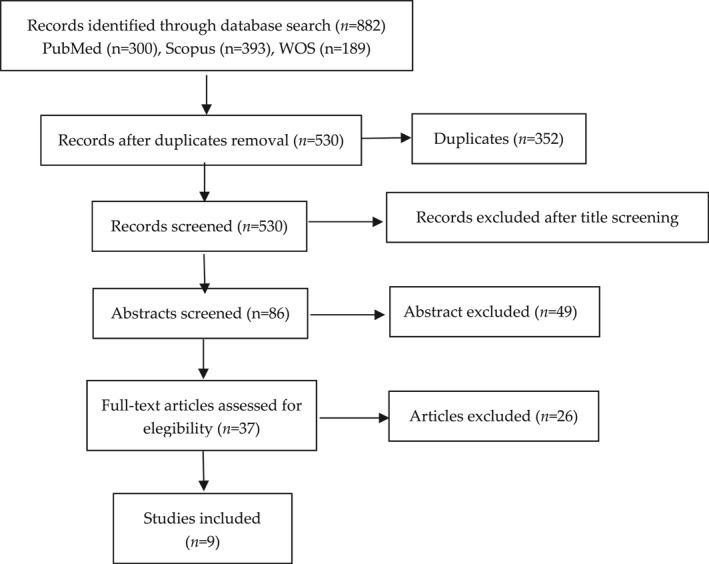
Flowchart of the selection process

**FIGURE 3:**
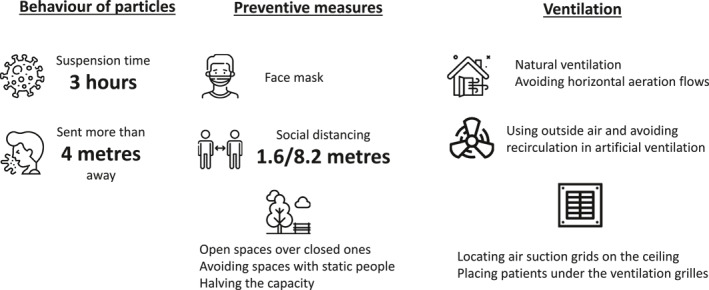
Main findings of the review

Initially, through the electronic search in the databases, 882 references were obtained. After eliminating duplicates, the titles and abstracts were analysed to identify the papers according to the inclusion criteria. The reading and analysis of full texts led to the selection of 9 papers for this review. The selection process is summarised in Figure 2. The main findings of the selected papers are presented in Table 1 and summarized in Figure 3.^20^, ^21^, ^22^, ^23^, ^24^, ^25^, ^26^, ^27^, ^28^

**TABLE 1:** Main results from the reviewed articles

Authors	Measured variables	Results	Recommendations
Bhagat et al. 2020	Ventilation.	Displacement ventilation, which encourages vertical stratification and removal of polluted hot air near the ceiling, appears to be the most effective measure in reducing contagion.	Ventilation that allows extraction of the columns of hot air in upper parts of rooms.
	Airflows.	Flows within enclosed spaces behave like turbulent.	Use of mask to send direct aerosols towards the body of the emitter, reducing the formation of polluting clouds.
	Wearing masks.	Masks are effective in reducing the direct expulsion of bio‐aerosols by dragging them into the person's body.	
Buonanno et al. 2020	Particle emission rate.	The viral load present in asymptomatic people is similar to that of those who present symptoms, so they have the same transmission capacity.	Use of effective protective measures since the contagion capacity of the asymptomatic is similar to the symptomatic.
	Ventilation.	The emission of particles when speaking resembles the emission that occurs when performing light physical exercise.	Mechanical ventilation in enclosed spaces.
		Mechanical ventilation reduces the number of cases that will be caused by an infected person during the period of infection from less than 1 to less than 0.4.	
Feng et al. 2020	Aerosol emission distance.	Coughing can send aerosols more than 3 m away if the air is stagnant.	Increase the safety distance in enclosed spaces without ventilation.
	Airflows.	The wind facilitates the movement and deposition of even large respiratory droplets.	Avoid direct air flows between individuals.
	Relative humidity.	High relative humidity increases the condensation effect and causes larger infectious droplets to fall earlier.	Increase the relative humidity in enclosed spaces.
	Wearing masks.	Incorrectly worn face masks also significantly reduce the suspension of contaminated particles.	Use face masks always and under any circumstances.
Riediker & Tsae. 2020	Viral load of aerosols.	An infected coughing individual emits approx. 10000 times more virus copies per cm^3^.	Use of face protection to reduce the risk of infection.
	Concentration in a closed room.	Concentrations in a closed space with an infected person coughing amount to 7.44 million copies/m^3^, while with normal breathing they represent 1248 copies/m^3^.	Ventilation of enclosed spaces.
Satheesan et al. 2020	Air conditioning systems.	Air renewal with the outside and the rates of exhaust airflow have significant effects on the distribution of polluting particles within a mechanically ventilated space.	Renewal with outdoor air in the air conditioning systems of indoor rooms.
	Air renewal.	The placement of air extraction grilles on top of an infected patient reduces the risk of airborne transmission.	Place infected patients under the air grilles.
Sun et al. 2020	Safety distance.	Safe social distance when speaking should be from 1.6 to 3.0 metres in ventilated or outdoor spaces, and up to 8.2 m in poorly ventilated environments.	Increase social distance as much as possible even in open spaces.
	Ventilation.	Decreasing the occupancy of spaces to 50% reduces the infection rate between 20% and 40% in the first 30 min.	Reduce space capacity to 50%.
		Safe ventilation of spaces reduces the risk by 40% if occupancy is reduced to 50%.	
Vuorinen et al. 2020	Number of particles inhaled for contagion.	Estimated number of particles for a contagion = 100.	Do not stay in enclosed spaces.
	Dispersion of aerosols.	Infectious aerosols remain up to 3 h in enclosed spaces.	Raise the safety distance to >4 m if there is coughing.
	Dilution time.	Ambient humidity decreases the suspension time.	Avoid spaces with people without movement (bars and offices).
	Ventilation.	Coughing sends infectious particles up to 4 m away in enclosed spaces and remain 90 s in the air.	Use suction ventilation from the ceiling.
		The speed of movement of the infected reduces the concentration of aerosols by dilution.	
		Suction ventilation from the ceiling favours the dilution of aerosols and reduces the risk of contagion.	
Yao et al. 2020	Humidity and temperature.	Both indoor and outdoor areas of high temperature and low humidity reduce the risk of infection.	Indoor spaces should increase temperature and reduce humidity to reduce the risk of infection.
	Use of ozone.	Ozone reduces the existence of the virus in all types of spaces and surfaces.	Use of ozone can help disinfect surfaces and environments.
			Adequately ventilate indoor spaces with outdoor air.
Zhao et al. 2020	Aerosol dispersion.	Respiratory droplets travel farther in environments with low temperature and high humidity.	Increase the safety distance to more than 2 m in humid and cold environments.
	Humidity and temperature.	The number of aerosol particles increases in high temperature and low humidity environments.	Avoid horizontal ventilations to aerate rooms, as these transport particles between people.
	Ventilation.	2 m of physical distance are insufficient to eliminate possible droplet contact in cold and humid environments.	
		The dispersion distance of the droplets increases as the speed of the airflow increases, which can reach 23 m..	

### 3.1 Behaviour of particles

Infectious aerosols are exhaled by infected people who emit a different number of particles, depending on whether they perform normal breathing or emit a cough or sneeze.^8^ According to the results by Buonanno et al.^21^ the viral load contained in aerosols emitted by symptomatic patients is similar to that emitted by those who are not. When considering the high number of people who have the disease but are asymptomatic, the risk of infection in any common space is high. Vuorinen et al.^26^ estimated at 100 the number of particles needed in the air for contagion. If the infected person is silent, the particle emission rate is 1248 copies/m,^3^ while if they speak they amount to 7.44 million copies/m^3^.^23^ When speaking, the emission rate is comparable to that of an individual doing light physical activity.^21^ According to Riediker & Tsae,^23^ an infected individual who coughs emits approximately 10000 times more virus particles/cm.^3^ In relation to the suspension time, Vourinen et al.^26^ established that the suspension time in closed places can reach 3 h.

Regarding the effect of coughing, several authors quantify its effect with respect to the displacement of infectious particles. According to Feng et al.^22^ these can be sent more than 3 m away in enclosed spaces and without ventilation. However, Vourinen et al.^26^ quantified this distance at 4 m and established an average critical stay in the air of 90 s.

### 3.2 Preventive measures

The most recommended containment measure is the use of a face mask, since regardless of the type used, it significantly reduces the contagion.^20^, ^22^ Bhagat et al.^20^ pointed out the protective effect of masks by diverting most of the particles emitted towards the infected person's own body. Feng et al.^22^ reinforced this recommendation by stating that even if they are poorly placed and made of low filtration material, they have protective effects against not wearing them.

**FIGURE 4:**
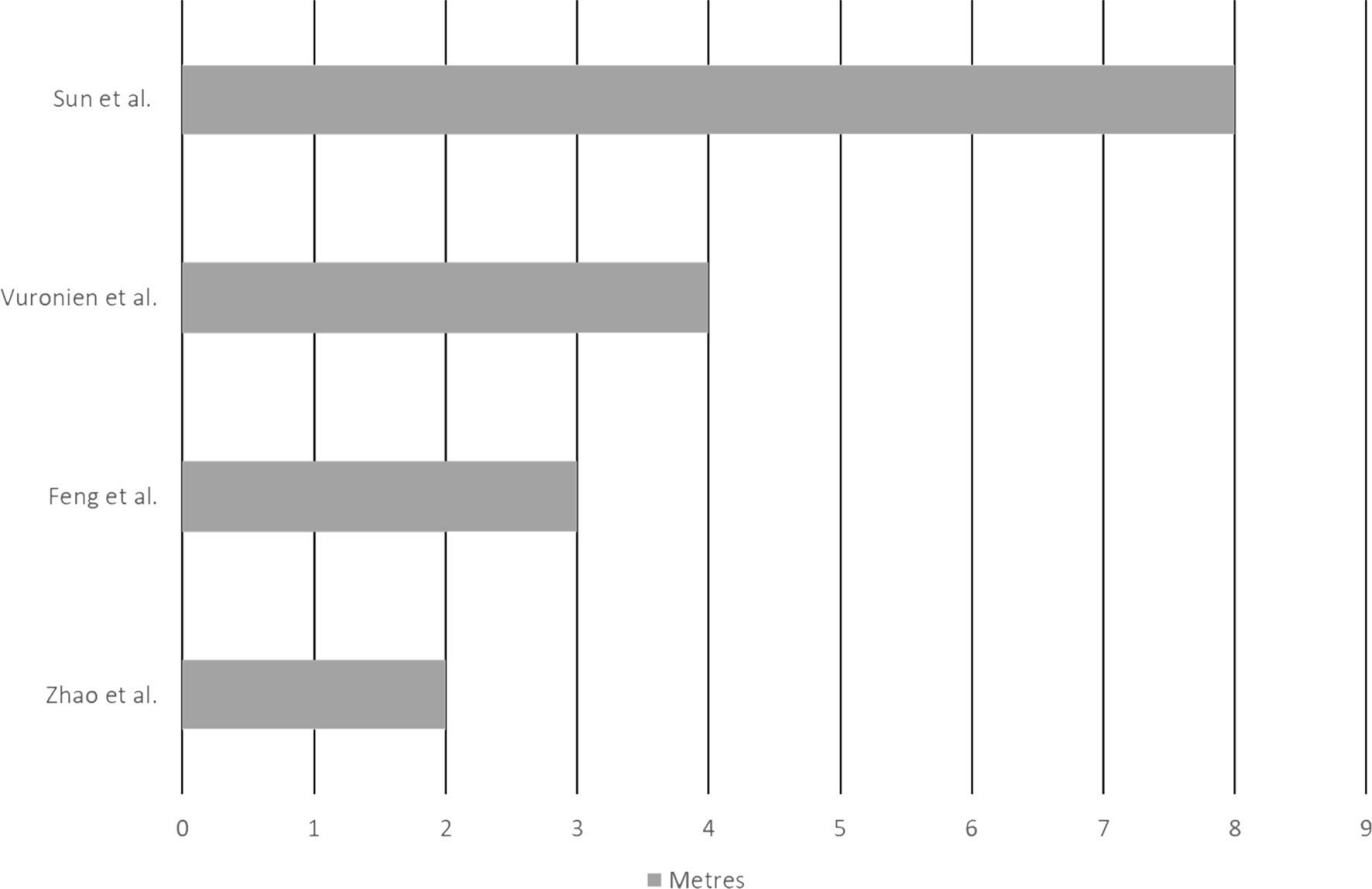
Recommended social distancing

With regard to social distancing measures, the revised articles established as an effective distance a minimum of 2 m even when wearing a mask,^28^ extending it to more than 4 metres in case of cough.^26^ In outdoor or ventilated spaces, between 1.6 and 8.2 m is considered a safe interval (Figure 4).^25^ In enclosed spaces, it is difficult to establish a safe range due to the difficulty of controlling influencing variables. It has been identified that an infected person in movement has a protective effect over standing in the same place, since the higher the speed of displacement, the greater the dispersion of aerosols and the lower the concentration.^26^

The difference in incidences of contagion varies depending on the characteristics of where social interactions occur and the capacity of enclosed spaces, so recommendations are established to promoting open spaces over closed ones,^21^, ^22^, ^23^, ^25^, ^28^ avoiding spaces with static people without movement,^26^ and halving the capacity to reduce the infection rate by 20%–40% in the first 30 min according to Sun et al.^25^

### 3.3 Ventilation

With regard to enclosed spaces, the characteristics of ventilation is the key to reducing the number of infections. Natural ventilation, with doors and windows to the outside, is more effective than artificial ventilation as it favours that larger particles are deposited earlier,^22^ and facilitates the dissolution of polluting aerosols with the clean air of the room.^24^ According to their results, Zhao et al.^28^ recommended avoiding horizontal aeration flows that transport particles from one person to another. On the contrary Buonanno et al.^21^ found that the effectiveness of mechanical ventilation is greater than natural ventilation in spaces visited by an asymptomatic patient, reducing the mean number of cases that will be caused by an infected person during the period of infection, from less than 1 to less than 0.4. According to these authors, if natural ventilation involves a direct flow of air from the infected person to the potential host, the chances of infection are higher.

As far as artificial ventilation is concerned, one variable to consider is the type of air circulation. The reviewed studies recommend the use of air from the outside and avoiding recirculation of indoor air to reduce the incidence of contagion.^24^ Bhagat et al.^20^ further described that moving airflows within enclosed spaces behave as turbulent, favouring the redistribution of potential polluting aerosols. Another variable to consider is the situation in which the air extraction device is placed in artificially ventilated rooms. According to the models included in this review, suction grids should preferably be located on the ceiling to be more effective.^20^, ^24^, ^26^ Specifically, in inpatient units of Covid‐19 patients, Satheesan et al.^24^ advised that patients should be placed under the grilles of the ventilation system to increase the suction power of infectious aerosols. Air extraction devices must be located in the highest areas of the rooms, so that the air that has risen due to temperature difference can be extracted.^20^

The reviewed studies also identified two other environmental variables that influence the contagion process: temperature and ambient humidity. The results suggest that rising temperatures reduce the risk of infection within an enclosed space (Yao et al., 2020; Zhao et al., 2020). As regards the ambient humidity, discrepancy is found in the results of the different studies. Some authors concluded that high relative humidity of the air favours longer air suspension of contaminating particles, which increases the risk of contagion.^27^, ^28^ On the contrary, other studies claim that high relative humidity reduces the risk of contagion from spaces where there is a Covid‐19 patient.^22^, ^26^ Finally, it is worth noting the importance of the use of ozone as a preventive method, since according to Yao et al. (2020) this compound reduces the permanence of SARS‐CoV‐2 on surfaces, reducing the risk of contact infection.

## 4 DISCUSSION

Our study aimed to describe the pattern of aerosol dispersion and its relationship to the transmission and contagion of SARS‐CoV‐2 in enclosed spaces. The results of the studies reviewed revealed that asymptomatic infected patients emit toxic aerosols in a similar way to those who are not asymptomatic. Breathing silently, talking, coughing, and sneezing emit particles with viral load in increasing order of concentration, speed, and distance reached. The authors agreed to recommend the mask as a measure to contain the disease, although they disagreed on the minimum social distance. In relation to ventilation, the studies included in the present review suggested natural ventilation as the most effective measure to prevent infection, and in the case of artificial ventilation, the use of outdoor air is recommended. Finally, the results revealed that a high temperature reduces the risk of transmission of the virus, but there was no consensus on the role played by relative humidity.

The results of the reviewed articles support the relevance currently given to the role of aerosols in the spread of SARS‐CoV‐2. WHO initially stated that the virus was not transmitted by air,^18^ based on the evidence available at the time.^3^, ^29^ With the passage of months and the increase in cases, research was developed aimed at better understanding the behaviour and spread of the virus. In light of the new results, health authorities changed their position on the route of infection, stating airborne transmission of SARS‐CoV‐2, as of 9 July, 2020.^30^

The behaviour of aerosols described by the reviewed studies is in line with previous studies. Kohanski et al.^12^ identified that if such particles are 1 μm in size, they can remain up to 9 h, and 39 days when smaller than 0.1 μm. Lipinski et al.^31^ determined that, when speaking, the largest saliva droplets move at a speed of 5 m/s, doubling in the case of coughing, and multiplying by four in case of sneezing. It has also been studied that aerosols carrying Covid‐19 pathogens can travel up to 7–8 m after a sneeze and more than 2 m (up to 4.5 m) after coughing.^32^

The studies included in this review reinforce the recommendation of the use of the mask as a measure of containment of the virus. These results coincide with previous authors^33^, ^34^, ^35^ who defend the mandatory nature of masks in public places as a mechanism for preventing infection. According to Oran and Topol,^36^ between 40% and 45% of Covid‐19 patients that are going through the disease are asymptomatic, so the use of masks is essential for controlling the transmission of the SARS‐CoV‐2. In spaces without identified positive patients, it is recommended to use a surgical mask,^33^, ^37^ and in the presence of an infected person, another type of filtration with greater power is recommended such as FFP‐2, FFP‐3, or N95 masks.^38^, ^39^ The effectiveness of masks is difficult to assess as their filtration capacity depends on the tissue used and whether or not filters are included.^40^

In relation to social distance, the reviewed studies support this measure. However, there are differences in the results of previous studies depending on the scenario. In ventilated spaces, if the infected person is at rest, a minimum separation between 1 m, according to WHO,^30^ and close to 2 m is recommended^34^, ^41^ as long as the infected person wears a face mask. Otherwise, these distances would be insufficient.^42^ In the event that the person is in a space without ventilation, the distances must be increased, being difficult to quantify due to the different variables that affect this situation.^43^

After reviewing the articles included in this review, ventilation stands out as a protective measure for the control of contagions in enclosed spaces, considering the possibility of contagious particles remaining in suspension. The advance of the pandemic and a better knowledge of the virus and the possibility of contagion through respiratory particles emitted by positive patients led to consider the effect of infectious aerosols as one of the most relevant vectors of contagion.^3^, ^43^ Aerosols are transported more quickly through the air when there are cross‐flows of air, and the permanence of the particles in the air is shortened when subjected to a significant downward current.^8^

Many studies propose natural ventilation as the most effective measure, that is keeping windows and doors open.^8^, ^37^ According to Kohanski et al.^12^ with three outdoor air renewals per hour in an enclosed space, the risk of contagion is reduced by 95%. If this is not possible, artificial ventilation is the next option, which would depend on the use of air conditioning devices.^44^

Evidence recommends placing the ventilation device on the ceiling, directed horizontally with respect to airflow, and just above the patient in a hospital with infected patients.^45^ Air recirculation would increase the permanence of aerosols in suspension, raising the risk of infection if there is a person infected in the enclosed space.^46^ Borro et al.^47^ recommend boosting clean air from the outside and avoiding recirculation, and if possible, the use of High Efficiency Particulate Arresting air‐purifying filters is recommended, as they remove the remains of the virus, thus preventing contagion.^48^, ^49^ Infectious particles have been found in ventilators in the air grilles of rooms with Covid‐19 infected patients, suggesting that aerosols contaminated with the virus have been displaced by airflows and deposited in the ventilation duct.^50^ Air conditioning systems often use filters that reduce the concentration of aerosols, eliminating more than 95% of particles of sizes between 0.3 and 10 μm in diameter.^51^

Although the reviewed studies identified that high temperatures delay the spread of the disease, there is controversy in determining the effect of relative humidity on the dissemination of the virus. On the one hand, authors affirm the enhancing effect of contagions when relative humidity is high.^52^ And, on the other, there are authors whose results disagree with this hypothesis.^53^, ^54^ Recently, Morris et al. conducted a study with a mechanistic model which revealed that SARS‐Cov‐2 live longer at low temperatures and extreme conditions of relative humidity, estimating a virus half‐life 22 h longer at 10°C and 40% relative humidity than at 27°C and 65% relative humidity.^55^

## 5 CONCLUSIONS

The present review brings together evidence on the behaviour of aerosols in the air, which will help to better understand the mechanisms of transmission of the SARS‐CoV‐2 virus. The results of the reviewed works confirm the airborne transmission route of the virus by means of contagious particles suspended in the air. These particles, aerosols, are emitted by infected people, symptomatic or not, during breathing activities such as breathing, talking, coughing, or sneezing. These particles have the property of remaining suspended in the air for prolonged periods of time, being greater when less air movement occurs in the area.

The reviewed articles support the implementation of three main contagion control measures: using masks as a method of reducing the volume of emitted respiratory particles; maintaining social distance with people of at least 1 metre, reducing the risk of infection the greater this distance is; and ventilating enclosed spaces, being preferable natural ventilation. In case of using artificial ventilation, the devices must be placed on the ceiling with horizontal airflow, avoiding the recirculation of air within the space.

Better knowledge of the virus will make it possible to design more effective infection control strategies. The findings gathered in this review are useful for the containment of the current pandemic and for future airborne diseases.

## AUTHOR CONTRIBUTION

José Miguel Robles‐Romero: Conceptualization, methodology, formal analysis, writing—original draft; Gloria Conde‐Guillén: Investigation, writing—original draft; Juan Carlos Safont‐Montes: Investigation, formal analysis, writing—original draft; Francisca María García‐Padilla: Investigation, formal analysis, writing—original draft; Macarena Romero‐Martín: Investigation, validation, writing—reviewing and editing, supervision.
